# Application of a TB care cascade to a novel household contact intervention in rural South Africa

**DOI:** 10.5588/pha.24.0031

**Published:** 2024-12-01

**Authors:** L. Linde, L.B. van Niekerk, K.W. le Roux, M. Wilson, M.B. Brooks, B.J. van de Water

**Affiliations:** ^1^ Boston University School of Public Health, Boston, USA;; ^2^ Zithulele District Hospital, Eastern Cape Department of Health, South Africa;; ^3^ University of Cape Town, Cape Town, South Africa;; ^4^ Advance Access and Delivery, Durban, South Africa;; ^5^ Boston College Connell School of Nursing, Chestnut Hill, USA.

**Keywords:** prevention, contact management, TB preventive treatment

## Abstract

Four clinics implemented an intervention to increase TB household contact identification and evaluation in rural Eastern Cape, South Africa. We applied a care cascade framework to assess gaps in evaluation and treatment initiation from April 2021 to June 2023. We identified 1,698 contacts of 287 individuals with TB. The majority of contacts (71%) were screened; 9% of those with symptoms were fully evaluated, and of these, 14% were diagnosed with active TB. This intervention substantially increased TB contact identification and evaluation compared to prior efforts in the same area; however, additional barriers limited the ability to identify and treat secondary cases.

## Abstract (fr)

Quatre cliniques ont mis en place une initiative visant à améliorer l'identification et l'évaluation des contacts familiaux de la TB dans la région rurale du Cap-Oriental, en Afrique du Sud. Nous avons utilisé un cadre de cascade de soins pour analyser les lacunes dans l'évaluation et le début du traitement entre avril 2021 et juin 2023. Au total, nous avons recensé 1 698 contacts de 287 patients atteints de TB. La majorité des contacts (71%) ont été soumis à un dépistage; parmi ceux présentant des symptômes, 9% ont bénéficié d'une évaluation complète, et parmi eux, 14% ont été diagnostiqués avec une TB active. Cette initiative a significativement amélioré l'identification et l'évaluation des contacts tuberculeux par rapport aux efforts précédents dans la même région ; néanmoins, des obstacles supplémentaires ont restreint la capacité à identifier et à traiter les cas secondaires.

The southern part of King Sabata Dalindyebo (KSD) sub-district of OR Tambo District, Eastern Cape, South Africa, is a rural area with limited health care access and high burdens of TB and HIV. In 2017, there was an estimated TB incidence of 840/100,000, with nearly half of patients with TB also being HIV-positive.^1^ Evaluation of close contacts of patients with TB (2018-2019) found that only 39% were screened for TB, with about 4% of those subsequently starting TB disease treatment and <1% starting TB preventive treatment (TPT).^1^

Household contacts (HHC) are a high-priority group for TB screening, as they are at increased risk for becoming infected and developing disease compared to non-close contacts.^2–4^ In 2019, the South African National Strategic Plan for TB proposed to include all HHC as eligible for TPT, expanding upon the existing strategy of TPT only for HIV-positive contacts or those under five years old; however, revisions were not published until 2023.^5,6^ In April 2021 the district hospital and three local clinics serving this area began implementing a novel intervention (KSD WIThout TB, or KWIT-TB) to improve HHC identification, case identification, and TPT uptake.

Care cascades are a useful framework to evaluate how individuals progress through disease identification and treatment stages, both for monitoring purposes and to identify gaps or barriers to successful care.^7^ We applied a care cascade framework to assess TB disease outcomes among the population identified by KWIT-TB screening efforts.

## METHODS

### KWIT-TB program

Field workers trained to the level of community health workers (CHW) visited patients diagnosed at the district hospital and three local clinics. They conducted one to two household visits for each individual diagnosed with TB. The CHWs identified HHC, ascertained HIV status, and conducted symptom screenings using the WHO 4-symptom screen in the home for those present. HHC with a positive symptom screen (at least one of cough, fever, night sweats, or unintentional weight loss) were referred to their local clinic for follow-up chest X-ray screening (if available), additional TB evaluation, and treatment initiation, if eligible. Phone calls were conducted at 12 months post-screening to assess post-treatment outcomes.

### Study design

**FIGURE 1.**
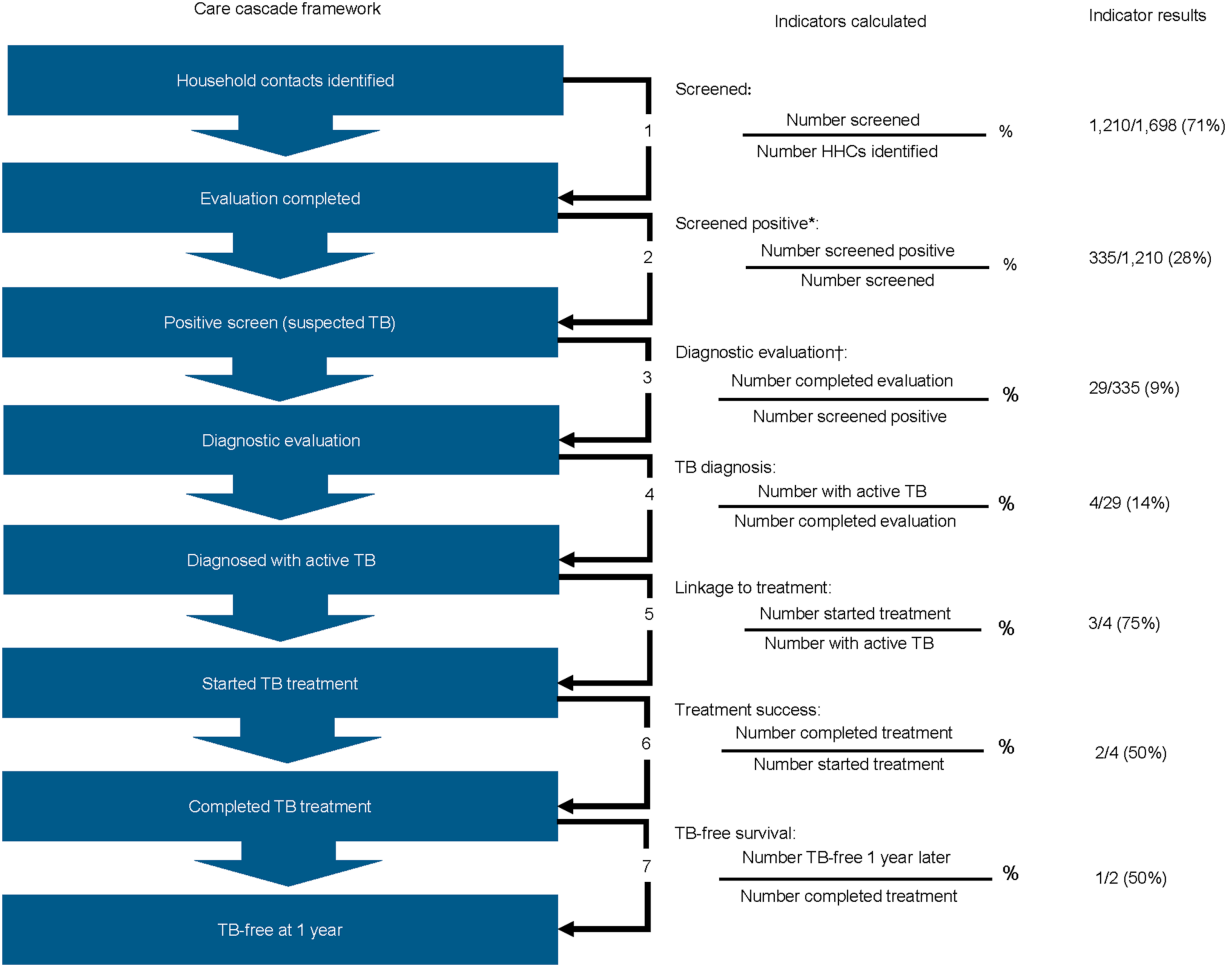
TB prevention care cascade framework, adapted from the Zero TB Initiative. Data were collected for each of the eight steps shown, and each of the seven indicators was calculated in percentages. *Screened positive: had at least one TB symptom (cough, fever, night sweats, unintentional weight loss). ^†^Diagnostic evaluation: GeneXpert testing (Cepheid, Sunnyvale, CA, USA) and/or chest X-ray. HHC = household contact.

We conducted a retrospective analysis of screening and medical chart data for HHC of individuals with TB identified by the four participating sites from April 2021 to June 2023. We adapted the TB treatment and prevention care cascades corresponding to the Zero TB Initiative’s ‘Search-Treat-Prevent’ schema^8^ to create an eight-step cascade (Figure 1).

### Data analysis

We used descriptive statistics to summarize HHC demographic and clinical characteristics. We evaluated the care cascade by assessing the following steps:

1 Step 1. The target population, the HHCs.
2 Step 2: The number of people who completed TB symptom screening.
3 Step 3: The number of people who screened positive.
4 Step 4: The number of people who completed the TB diagnostic evaluation with X-ray and/or GeneXpert testing (Cepheid, Sunnyvale, CA, USA).
5 Step 5: The number of people diagnosed with active TB.
6 Step 6: The number of those who started on TB treatment.
7 Step 7: The number of those who completed TB treatment.
8 Step 8: The number of those who were alive and free from TB symptoms and/or disease after one year.

We calculated the proportion of individuals moving between each step of the cascade overall and stratified by sex, age, HIV status, and clinic. Differences in the proportion of HHC moving between steps by group were compared using chi-square tests.

### Ethics

Institutional review board approval was provided by Walter Sisulu University, Mthatha, Eastern Cape, South Africa (024/2020), Harvard Medical School, Boston, MA, USA (IRB19-1739), Boston College, Chestnut Hill, MA, USA (22.060.01e), and the Eastern Cape Department of Health, Bisho, Eastern Cape, South Africa (EC_202006_006). No informed consent was obtained as care was provided as part of programmatic TB care at the four sites.

## RESULTS

We identified 1,698 HHC from 287 individuals with TB, averaging 5.9 HHC per patient. Among contacts, approximately half were female (52%), the average age was 22 years, with 15% less than five years old, and 17% of contacts were known to be HIV-positive.

Of the identified contacts, 1,210 (71%) were screened for TB (Figure 1). Of those screened, 335 (28%) reported at least one symptom, but only 29 (9%) were subsequently evaluated for TB. Four of the individuals (14%) were diagnosed with active TB disease, all of whom were female and over age 15, and one was HIV-positive.

**FIGURE 2.**
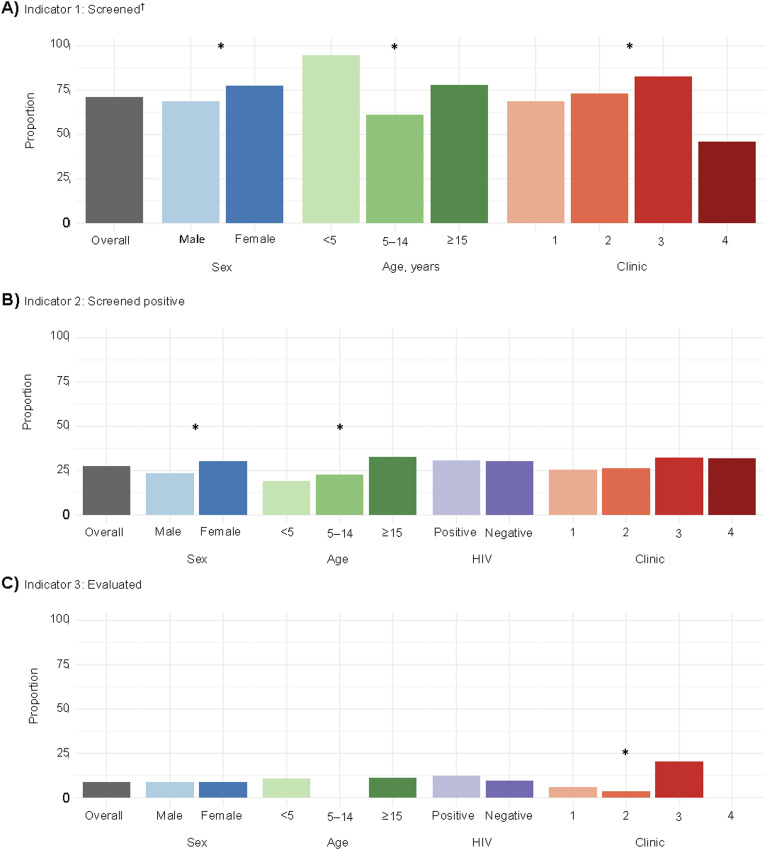
TB prevention care cascade outcomes for the first three of the calculated indicators, overall and stratified by demographic factors. *Statistically significant difference (*P* < 0.05) in indicated category using the χ^2^ test. ^†^HIV status not included in the screening cascade as HIV testing was part of the screening process.

More children under five years completed symptom screening compared to older ages (*P* < 0.001; Figure 2); however, more contacts over 15 years screened positive (*P* < 0.001). Clinic 3 screened the greatest proportion of contacts (*P* < 0.001) and completed the greatest proportion of diagnostic evaluations of those that screened positive (*P* < 0.001); three of the four individuals diagnosed with TB were from Clinic 3.

Of the 4 individuals diagnosed with TB, 2 completed treatment, 1 stopped treatment before completion, and 1 was lost to follow-up. Of the two that completed treatment, both were alive and disease-free after one year; one was asymptomatic, and the other reported a cough.

## DISCUSSION

Over a 2-year period, the KWIT-TB intervention substantially improved TB contact identification and evaluation compared to prior findings in the area.^1^ This 32-percentage point increase in household screening - from the previous 39% to the current 71% - provides insight into the potential impact of a systematic and concerted active case-finding effort in a defined community. While most contacts identified were screened, there remained a significant drop off from screening to completed TB evaluation among symptomatic contacts. Of note, for this study, we defined completing a TB diagnostic evaluation as having had an X-ray and/or GeneXpert, but more individuals were clinically evaluated by two doctors in the program. The improvement in screening achieved by the KWIT-TB team can be attributed to staff conducting active household contact tracing, visiting the most remote and inaccessible homes on foot and visiting some households up to three times to screen all potential contacts. Therefore, most of the burden was put on the health system to seek out contacts, making screening more accessible and family-centered.^9,10^ Another effective strategy was to call the households (via patient cell phone numbers) a few days before a planned visit. This allowed for household planning supporting screening activities to be completed efficiently and effectively.

However, once an individual was found to be symptomatic and needing additional examination, that person had to visit the health facility during regular facility hours. The KWIT-TB program often provided direct transportation when possible; however, transportation to facilities was a major barrier to care. Unfortunately, we did not measure stigma, which is repeatedly supported in TB literature as a major patient-related barrier in active case-finding programs.^11^ At this step of the cascade, the burden changed from being on the health system to being on the family or individual, likely causing the large drop-off. Supporting patient- and family-centered TB care and reducing the onus on individuals is paramount to close gaps in the TB care continuum, specifically the diagnostic gap.^12^ Additionally, Pai and colleagues outline seven transitions to specifically close the diagnostic gap – the largest gap in the TB cascade.^13^ The potential of home-based point-of-care molecular diagnostic testing has recently been piloted in the Eastern Cape, South Africa, providing evidence of acceptability and feasibility in rural and hard-to-reach settings.^14^

Individuals over 15 years were most likely to screen positive in our study; however, they were less likely than children aged <5 years to be screened. Our high screening rate among young children (95%) is an important success, as children aged <5 years are at high risk for developing TB and serious TB sequelae.^15^ This high screening rate among children aged <5 years may be due to younger children often being at home with caretakers, frequently grandparents, while older children are usually at school and individuals aged >15 years may be working harder to find at home during daytime household contact tracing visits. Pragmatic approaches are necessary to address screening and diagnostic gaps for all ages, especially adolescents, who are often the hardest to screen, particularly in low-resource settings.^16^ To increase screening rates for individuals typically out of the household during the week, the KWIT-TB program did operate on some weekends, although this was limited due to staff constraints.

Among individuals who completed TB evaluation, we found a relatively high rate of disease (14%) compared to prior assessments of the yield of household contact investigations in low- and middle-income countries, but absolute numbers were very small.^4^ If this rate of disease was reflected in the overall group of symptomatic contacts identified, crude estimation indicates complete TB evaluation of this group may have yielded an additional 42 cases. While the KWIT-TB program reduced barriers to identifying household contacts and initiating TB screening, the additional step of TB evaluation remained clinic-based and logistically challenging, ultimately limiting the ability to identify and treat secondary cases.
